# Bridges, brokers and boundary spanners in collaborative networks: a systematic review

**DOI:** 10.1186/1472-6963-13-158

**Published:** 2013-04-30

**Authors:** Janet C Long, Frances C Cunningham, Jeffrey Braithwaite

**Affiliations:** 1 Centre for Clinical Governance Research, Australian Institute of Health Innovation, University of New South Wales, Kensington, Australia

**Keywords:** Brokerage, Collaborative networks, Structural holes, Social network theory, Knowledge transfer

## Abstract

**Background:**

Bridges, brokers and boundary spanners facilitate transactions and the flow of information between people or groups who either have no physical or cognitive access to one another, or alternatively, who have no basis on which to trust each other. The health care sector is a context that is rich in isolated clusters, such as silos and professional “tribes,” in need of connectivity. It is a key challenge in health service management to understand, analyse and exploit the role of key agents who have the capacity to connect disparate groupings in larger systems.

**Methods:**

The empirical, peer reviewed, network theory literature on brokerage roles was reviewed for the years 1994 to 2011 following PRISMA guidelines.

**Results:**

The 24 articles that made up the final literature set were from a wide range of settings and contexts not just healthcare. Methods of data collection, analysis, and the ways in which brokers were identified varied greatly. We found four main themes addressed in the literature: identifying brokers and brokerage opportunities, generation and integration of innovation, knowledge brokerage, and trust. The benefits as well as the costs of brokerage roles were examined.

**Conclusions:**

Collaborative networks by definition, seek to bring disparate groups together so that they can work effectively and synergistically together. Brokers can support the controlled transfer of specialised knowledge between groups, increase cooperation by liaising with people from both sides of the gap, and improve efficiency by introducing “good ideas” from one isolated setting into another.

There are significant costs to brokerage. Densely linked networks are more efficient at diffusing information to all their members when compared to sparsely linked groups. This means that while a bridge across a structural hole allows information to reach actors that were previously isolated, it is not the most efficient way to transfer information. Brokers who become the holders of, or the gatekeepers to, specialised knowledge or resources can become overwhelmed by the role and so need support in order to function optimally.

## Background

Bridges, brokers and boundary spanners facilitate transactions and the flow of information between people or groups separated or hindered by some gap or barrier. This may be a physical gap such as geographic location, cognitive or cultural gap such as differing disciplines or professions or alternatively, the gap may be that members of one party have no basis on which to trust the other. Studies on brokerage have included large, distributed or geographically separated organisations and corporations, commercial settings with diverse markets, political networks, affiliations and partnerships. The health care sector is another context that is rich in isolated clusters in need of connectivity such as silos, professional “tribes,” and clinical versus managerial domains [1-3]. Brokerage is therefore of particular interest in this context and lessons learnt in other collaborative settings may be useful. It is a key challenge in health service management to understand, analyse and exploit the role of key agents who have the capacity to connect disparate groupings in larger systems [4].

Early theory on these types of roles was developed in Burt’s *Structural holes*[5], and his later book *Brokerage and Closure*[6] placing them firmly in the context of social network theory. Networks are increasingly seen as an optimal structure via which to both organise, and think conceptually about, clusters of diverse individuals, groups or organisations who aim to work together collaboratively [7,8]. The network approach focuses on the relationships and interactions of the members (actors) rather than their individual attributes or behaviours. Key underpinnings of networks are that they are composed of nodes (the actors in the network) and ties (the relationships between actors). The ties form the structure of the network and the nodes occupy positions within that structure [7]. Bridges, brokers and boundary spanners hold key structural positions in networks affording opportunities and constraints on their actions. Social network analysis (SNA) techniques allow these actors to be identified and the structure of the network to be empirically described, graphed and analysed [9]. Network analysis can provide information on such processes as communication flows and bottlenecks, which in turn may suggest interventions to enhance function [10]. Recent reviews on network theory and practical applications are Chauvet [10], Borgatti and Halgin [7] and Kilduff and Brass [11].

Bridges, brokers and boundary spanners are just three of the most common descriptors in a lengthy list of synonyms for these roles reflecting the highly nuanced nature of the connectivity function. We follow Burt [5] by using the term brokerage to refer generally to the position. Brokers are said to reach across a structural hole. A structural hole manifests between two actors that are said to be non-redundant: that is between two actors who themselves are not connected [5]. Brokerage provides benefits for the individual based on the idea that non-redundant actors are sources of unique information that can be used by the broker for personal advantage by increasing their social capital [5]. Social capital is defined as the advantage created by a person’s location in a structure of relationships [6,12] and contrasts with the idea of human capital [5,13] which explains a person’s advantage in terms of personal attributes. Brokers can facilitate access to novel information, or resources, facilitate transfer of knowledge, and co-ordinate effort across the network. Boundary spanning as a form of brokerage includes the idea of crossing organisational boundaries such as departments or organisations [14], or cultural boundaries such as disciplines [15] in order to exchange knowledge or mediate interactions. Brokers are considered key players in that their loss from a network would greatly affect its function and viability [16]. Table 1 summarises the use of key brokerage terms in network theory.

**Table 1:** Common brokerage terms, features and motivations.

**Term**	**Features**	**Motivation**	**Reference**
Boundary spanner	Bridges the structural hole between two clusters conceptualised as being separated by a boundary of some sort, e.g. outside the network or department	To overcome a boundary and facilitate communication / knowledge flow across it.	[17,18]
Bridge	Bridges the structural hole between two clusters	To include outsiders in information flows or achieve coordination.	[5,19]
Broker	Acts as an intermediary between two unlinked actors / clusters	To facilitate some transaction, resolve a conflict or increase personal power or social capital.	[18,20-22]
Broker in a Structural fold	The broker is the common actor in two overlapping, cohesive clusters	To be an engaged member of two groups. Tends to be disruptive as loyalties may be seen to be divided.	[23]
Consultant / cosmopolitan / itinerant broker	Links two alters in an outside cluster/s who are not directly linked	To facilitate negotiations between alters or seek to exploit their separation.	[21,22]
Co-ordinator	Links alters within their own cluster who are not linked directly	To improve coordinated effort or to centralise knowledge exchange.	[21,22]
Gatekeeper	Bridges the structural hole between their cluster and an outside cluster, controlling what information passes into or out of their cluster	Often associated negatively with a hoarding of information, or positively bringing useful information / filtering out irrelevant information.	[21,22,24]
Go-between	Stands between two unlinked actors offering some service, e.g. facilitating access to advice, resources	Usually facilitative but can result in work overload for actor or information bottlenecks.	[25,26]
Information or knowledge broker	Keeps separate groups in a network co-ordinated or informed	To improve network information flows and prevent fragmentation.	[18]
Liaison	Bridges the gap between two different outside clusters without having prior allegiance to either	To facilitate negotiations between alters – often a commercial transaction.	[21,22]
Mediator / conflict resolver	Seeks to increase understanding between two parties separated by a mismatch of knowledge, expectations, culture etc.	To resolve conflict between parties - role often held by actor familiar with both sides.	[15,27,28]
Peripheral specialist	Holder of specialised knowledge that tends to occupy peripheral positions	To be available for consultation yet still devote time to their specialty.	[18]
Representative	Bridges the gap between another actor from the same cluster and an actor from an outside cluster	To facilitate external contact - may be a delegated negotiator.	[21,22]
*Tertius gaudens* (the third who enjoys)	A brokerage strategy to keep alters apart	To increase broker’s personal social capital or power.	[5,27]
*Tertius iungens* (the third who joins)	A brokerage strategy to join alters together	To increase network performance.	[29,30]

The aim of this review is to systematically review the empirical, peer reviewed research on bridges, brokers and boundary spanners from a network theoretical perspective, across a wide range of collaborative settings in order to inform our understanding of brokerage in networks. Most research is outside of health care, although there is increasing interest from health services and clinical researchers on network roles. Thus, we draw on a wider range of studies, including non-health research, to examine this issue.

## Methods

The literature search was conducted in December 2011, over the period January 1994 to November 2011 following PRISMA guidelines [31]. The year 1994 was chosen to coincide with published research on brokers arising from Burt’s book, *Structural holes*[5]. Databases used were: Medline, CINAHL, Business Source Premier (BSP), and the International Bibliography of Social Sciences (IBSS). We also undertook pilot searches of Web of Science, ERIC and PsychInfo but they did not yield any additional articles. Search terms were chosen on the basis of scrutiny of the literature, exploration of MeSH terms and terms suggested by expert researchers in the field. The lack of standard key terms for social network theory was problematic. “Social network” for example tended to be used synonymously with social media (such as Facebook) or social support. By looking specifically for terms used in social network analysis (e.g. betweenness and centrality) within the title and abstract we were able to increase our yield. In spite of this, we were aware of a number of relevant articles that were still not picked up in the searches, so we used a snowball process to generate an additional list of 91 articles.

**Figure 1:**
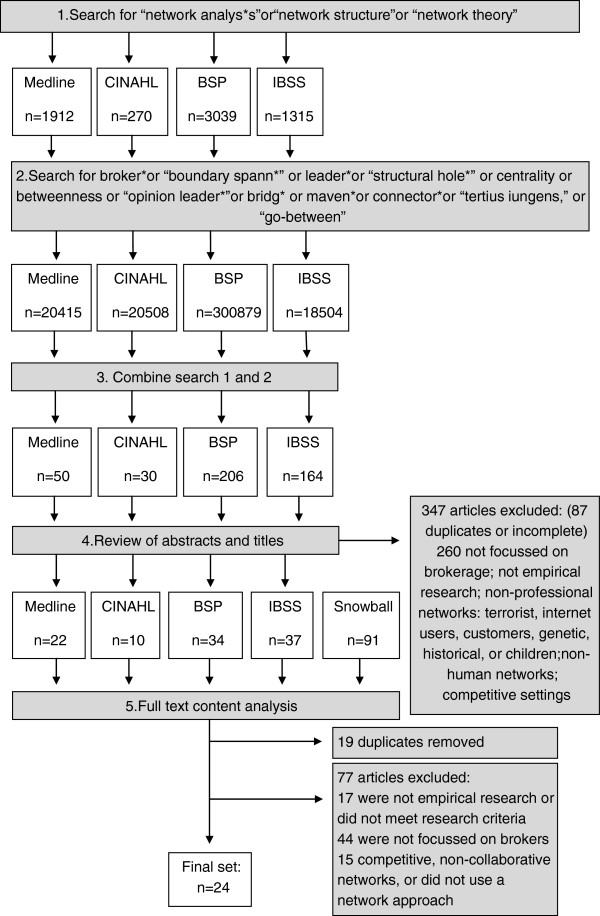
Flow chart of search strategy for literature review.

Articles were downloaded into Endnote X5, a bibliographic database. Duplicates and incomplete references were discarded. Two reviewers, (JL and FC) analysed titles and abstracts and removed articles if they were not empirical research articles (e.g., opinion pieces, reviews, theoretical papers), were not in English, or did not meet the other inclusion and exclusion criteria (Table 2). The papers’ empirical assessment of aspects of the brokerage role was a primary criterion. Articles that considered leaders (with high centrality) who were also brokers were only included if the brokerage role was the focus. One of our inclusion criteria was that networks needed to be collaborative in intention, not exploitative or competitive. This meant that some commercial settings were included and some excluded. Articles were also assessed using more detailed research quality criteria (Table 3). Criteria included a clear description of participants, context, data collection and analysis methods. Articles were included or excluded after discussion and agreement between two of the authors (JL and FC). The final step of the process was a content review of the full articles by one author (JL) with summary data being compiled in a table: study objective, context, participants, study design, analysis, method used to identify brokers, and findings. All authors reviewed this documentation for accuracy and completeness. The full search strategy is outlined in Figure 1.

**Table 2:** Inclusion and exclusion criteria for articles

**Inclusion criteria**	**Exclusion criteria**
Empirical research on brokers, bridges or boundary spanners within a collaborative network using a network approach	Not empirical research, e.g. models, concepts, methods, frameworks, tools, computational or theoretical aspects of network theory or collaboration
Social network of professionals e.g. health, academic, research, corporate, commercial	Social networks of non-professionals such as students, children, internet site users, genetic or disease groups, terrorists or criminals, historical groups, families, friends, local community members, targets for health promotion or marketing, customers or recommender groups
Local or virtual means of interaction	Non-human social networks (e.g. animal societies, molecular systems) or simulations of human networks
Individual, organisational or interorganisational level data	
Brokers, bridges or boundary spanners identified sociometrically or ethnographically	

**Table 3:** **Detailed research criteria (from Table S3 in**[32])

**All study designs:**	**Ethnographic studies:**	**Social network studies:**
Appropriate research question	Description of study setting and context	Network boundaries clearly defined
Details of study design given	Description of study methods	Level of analysis defined
Description of sources for data collection	Adequate number of participants	Response rates given for whole network surveys
Survey techniques described	Adequate observation period	Clear definition of tie relationships, direction and strength
Description of analysis	Means of identifying brokers clearly defined	Appropriate means for identifying brokers
Data presentation		Description of analysis
Discussion of results		
Study conclusions		
Clear definition of tie relationships, direction and strength		
Appropriate means for identifying brokers		
Description of analysis		

## Results

### General characteristics

The 24 articles that made up the final literature set were from a wide range of settings and contexts: from Italian Television production teams to Australian hospitals, Scandinavian telecommunication companies to US-Indian engineering projects. This enabled us to describe a rich picture of the brokerage phenomenon. Table 4 summarises the articles’ context, methods and findings on bridging, brokering or boundary spanning positions. More articles were generated in the last six years: 18 articles published between 2005 and 2011, while only six were from the decade 1994 to 2004. Nineteen of the 24 articles cite Burt’s seminal work on brokerage roles, *Structural holes*[5], and articles written in the last decade also cite *Brokerage and closure*[6]. Three studies not referring to Burt’s work are focused on boundary spanning and draw on different theoretical antecedents such as work by Tushman on boundary roles in the innovation process [17], Friedman and Polodny’s work on role conflict among boundary spanners during labour negotiations [14] and Allen’s work on ‘technological gatekeepers’ in R&D laboratories [33].

**Table 4:** Summary of included articles’ study design, context, method of identifying brokers and key findings about brokers

**Authors, date**	Study design*	**Brokers identified by**	**Context, settings**	**Findings about brokers**
Ahuja, G. (2000) [34]	1. Interorganisational	Nonredundant contacts per total contacts	Firm collaborations within the international chemicals industry	Brokering structural holes between companies increases innovative output up to a point before it decreases.
	2. Longitudinal, retrospective			
	3. Documentary data			
	4. Regression analyses			
Aral, S. & Van Alstyne, M. (2011) [35]	1. Interpersonal	Network constraint	Employees from a US executive recruiting firm	Brokers’ success at accessing novelty depends on their knowledge environment.
	2. Cross-sectional			
	3. Analysis of email content			
	4. SNA, word mining			
Balkundi, P., Barsness, Z. et al. (2009) [36]	1. Interpersonal	Betweenness centrality	19 teams from across two US paper and wood-based building product plants	Leaders who were brokers (high betweenness centrality) in the advice-seeking network had teams with higher team conflict and lower viability.
	2. Cross-sectional			
	3. Paper-based survey using roster			
	4. SNA			
Bercovitz, J. & Feldman, M. (2011) [37]	1. Interpersonal	Measure of "expertise distance" between academic departments; number of ties to external networks	Academic research teams from two US universities	Costs are involved in coordinating diverse teams but such teams are more successful inventors.
	2. Cross-sectional			
	3. Documentary data: invention disclosures, personnel records, patents			
	4. PROBIT modelling			
Burt, R. (2004) [12]	1. Interpersonal	Network constraint	US electronics company managers	Brokers accrue social capital by being able to see and express more “good ideas.”
	2. Longitudinal, retrospective			
	3. Online survey; archival data			
	4. SNA; regression analyses			
Colazo, J. (2010) [38]	1. Interteam	Boundary-spanning activity (number of team members who work on another project per number of members in focal team)	Open source software development teams	Boundary spanning activity in teams was positively associated with quality but negatively associated with productivity.
	2. Longitudinal, retrospective			
	3. Archival data on teams and project quality			
	4. SNA, regression analyses			
Creswick, N. & Westbrook, J. (2010) [39]	1. Interpersonal	Betweenness centrality	Communication between ward staff of an Australian teaching hospital	SNA can identify strategic people that act as brokers.
	2. Case study			
	3. Paper-based survey using roster			
	4. SNA			
Cummings, J. & Cross, R. (2003) [25]	1. Interpersonal	Effective size	182 work groups (average 8 members) in a US Fortune 500 telecommunication firm	Leaders who act as brokers ("go-betweens") within teams can cause a bottleneck in information flow that can decrease productivity.
	2. Cross sectional			
	3. Email survey using roster			
	4. Regression analyses			
Di Marco, M., Taylor, J. et al. (2010) [28]	1. Interpersonal	Betweenness centrality	Indian and US post-graduate students in two engineering project teams	Nominated cultural boundary spanner (CBS) can decrease cultural based knowledge system conflicts and trigger emergent CBS.
	2. Ethnographic			
	3. Observation over 3 days			
	4. SNA			
Fleming, L., Mingo, S. et al. (2005) [40]	1. Interpersonal	External ties (ln)	35,400 inventors across 16 East German regional innovation networks	Brokers can generate innovative ideas but their presence can hamper its diffusion and use.
	2. Longitudinal, retrospective			
	3. Archival patent data			
	4. Regression analyses			
Hanson, D., J. Hanson, et al. (2008) [41]	1. Interpersonal	Betweenness centrality	152 members of an Australian network of community groups for safety promotion	Asymmetric distribution of influence: six members with high centrality and betweenness centrality.
	2. Longitudinal case study, prospective			
	3. Paper-based survey; 3 initial waves of snowballing to identify members			
	4. SNA			
Hargadon, A. & Sutton, R. (1997) [42]	1. Interpersonal	Observation	Design engineers at IDEO, a US product design firm	Technology brokering involves four stages: access, acquisition, storage and retrieval.
	2. Ethnographic			
	3. Observation, interviews			
	4. Grounded theory			
Hawe, P. and L. Ghali (2008) [43]	1. Interpersonal	Betweenness centrality	Staff and teachers at a Canadian high school	SNA useful tool to identify people of strategic influence (including brokers) in health promotion activities.
	2. Case study			
	3. Paper-based survey using roster			
	4. SNA			
Heng, H. K., W. D. McGeorge, et al. (2005) [44]	1. Interpersonal	Betweenness centrality; effective size and efficiency (SH)	Department managers of an Australian hospital	Facility manager had high brokerage potential.
	2. Case study			
	3. Survey using name generator			
	4. SNA			
Lingo, E. & O'Mahony, S. (2010) [29]	1. Interpersonal	Observation; assessment of *tertius* orientation (*tertius gaudens or tertius iungens)*	Independent music producers in the Nashville (US) country music industry	Brokerage is a process (cf. position) and both *tertius* orientations can be used to produce collective outcomes.
	2. Ethnographic			
	3. Observation, interviews			
	4. Grounded theory			
Luo, J.-D. (2005) [26]	1. Interpersonal	Betweenness centrality	296 workers in two multinational technology companies in mainland China and in Taiwan	Brokers ("go-betweens") in advice-seeking networks have informal power and are higher in particularist trust than others.
	2. Cross-sectional			
	3. Survey			
	4. Regression analyses			
Marrone, J., Tesluk, P. & Carson, J (2007) [45]	1. Interpersonal	Self- and alter-assessment	190 MBA students in 31 teams in a US university consulting project	Team level boundary spanning mitigates the negative cost of individual boundary spanning.
	2. Cross-sectional			
	3. Survey			
	4. Hierarchical linear modelling (individuals nested within teams)			
Obstfeld, D. (2005) [30]	1. Interpersonal	Constraint; *tertius iungens* orientation	Designers, engineers and managers in a US engineering division of automotive manufacturer	*Tertius iungens* orientation, social knowledge and network density are independent predictors of involvement in innovation.
	2. Ethnography, case study			
	3. Email survey using name generator, interviews, observation			
	4. Qualitative, regression analyses			
Padula, G. (2008) [46]	1. Interorganisational	"Shortcuts:" number of cumulative alliances to other clusters	US mobile phone firms	Network cohesion and brokerage ("shortcuts") synergise to produce best environment to generate and produce innovation.
	2. Longitudinal, retrospective			
	3. Archival patent data			
	4. Regression analyses			
Rangachari, P. (2008) [47]	1. Interpersonal	Between subgroups in structural equivalence analysis	Administrators and professional staff from four hospitals in New York State	Brokerage across professional subgroups results in better coding performance.
	3. On-line survey using roster; interviews			
	4. SNA; structural equivalence analyses			
	2. Cross-sectional			
Rodan, S. & Galunic, C. (2004) [48]	1. Interpersonal	Network sparseness = 1-Density	Managers from a Scandinavian telecommunications company	Access to heterogeneous knowledge may be more important than sparse network structures for innovative managerial performance.
	2. Cross-sectional			
	3. Paper-based surveys using roster and one wave of snowballing to include named external contacts			
	4. Regression analyses			
Soda, G., A. Usai, et al. (2004) [49]/ Zaheer, A. and G. Soda (2009) [50]	1. Interpersonal then aggregated to team level	Network constraint	TV production specialist teams from Italy	Current brokerage associated with higher team performance. Past brokerage ties are not as effective as current ones.
	2. Longitudinal, retrospective			
	3. Archival data on 501 TV			
	productions			
	4. SNA, regression analyses			
Susskind, A., P. Odom-Reed, et al. (2011) [51]	1. Interpersonal	Network constraint, effective size, efficiency and hierarchy	Members of 11 hospitality management programs across six hotels and 11 US universities	Level of brokerage was not significantly related to individual team member performance but negatively related to overall team performance.
	4. SNA, regression analyses2. Cross-sectional			
	3. Survey using roster			
Tiwana, A. (2008) [52]	1. Interpersonal	"Bridging ties" extent of heterogeneity of expertise, background and skills of fellow team members	173 team members within a US internet business applications company	Both strong ties and brokerage (“bridging”) ties are needed to realise knowledge integration.
	2. Cross-sectional			
	3. Survey			
	4. Regression analyses			

*Study design legend: 1. Level of analysis (nodes as individuals, teams or organisations); 2. Design; 3. Method of data collection 4. Method of analysis.

Levels of analysis differed. While the majority used individuals as the nodes describing relationships, two studies were at the interorganisational level looking at collaborations across an inter-firm alliance (the firms were the nodes). Eleven studies looked specifically at the interpersonal relationships within well-defined teams or project groups of less than 12 members. Some studies aggregated individual data from the teams to a single team score.

### Data collection and analysis

Methods of data collection varied with ten using surveys and seven using archival documentary data such as patents, emails or personnel records to record relational ties and attributes of the participants. Ethnographic methods such as observing interactions and interviewing participants were used in four studies. The majority of studies used regression analyses to test the impact of their chosen variables on brokerage. For example, Susskind and colleagues [51], testing the variable “team performance,” were able to ascertain that overall team performance in their study was negatively associated with more brokerage activity. Fifteen studies used SNA to compute network characteristics.

There was a marked difference in the way bridging, brokering or boundary-spanning positions were identified. Seven studies used Burt’s network constraint measure (or a measure derived from it), [5] which is used to reflect brokerage potential around a particular actor. It varies with the size, density and hierarchical nature of the network. High constraint means the actor’s egonet (the ties of that actor alone) has few nonredundant contacts, while low constraint has many. Eight studies used the algorithmic parameter betweenness centrality [53] that measures the extent to which an actor lies between other actors that would not otherwise be connected (that is are nonredundant). Seven studies used straight frequencies of external or bridging ties and four used direct observation or self-assessment by actors and their peers or supervisors.

### Identifying brokers and brokerage opportunities

We found four main themes addressed in the set of 24 articles. There were four studies that took the benefits of brokerage as a given and sought only to confirm brokerage opportunities in a particular network, or identify individuals with high brokerage potential [39,41,43,44]. All four used betweenness centrality to identify the brokerage positions and the intention was to maximise efficient knowledge transfer, co-ordinate effort or to ensure the inclusion of people on the periphery. All four were based in a hospital or health promotion setting.

### Generating and integrating innovation

There were 12 articles that sought to define the network conditions and contexts in which brokerage facilitated the generation and integration of innovative ideas [6,29,30,34,35,37,40,42],[46,48,49,52]. The interplay of brokerage opportunities and network cohesion in this process of innovative performance, referred to by Burt [6] as brokerage and closure, is the main focus of seven of these articles.

Schumpeter’s [54] concept of innovation as the recombination of diverse understandings and knowledge is often cited to explain how brokerage can generate innovation. Burt [12] called it the “vision advantage:” the ability to select and synthesise different viewpoints and approaches from different, unlinked groups on either side of a structural hole. Hargadon and Sutton’s [42] ethnographic study of design engineers at IDEO, a US design firm, explored this process. The researchers saw the company itself as the broker embedded in a broad network of industries where there were gaps (structural holes) in the flow of technological knowledge. IDEO’s product designers deliberately brokered those gaps by looking for technological solutions from one industry that could be applied successfully to another, often resulting in an innovative, recombinant solution. For example, they described how the project team looking for a better design for a surgical skin stapler considered powering it with a gas engine from a model aeroplane.

“Good ideas” may involve declarative or factual knowledge, as well as tacit knowledge that grows up around highly specific contexts. However, while networks rich in structural holes are ideal to generate innovative ideas, they are the least well suited to integrate those ideas. The diffusion of innovations literature suggest the optimal network structure for spreading factual and especially tacit knowledge is cohesion (high density) not sparseness (more structural holes) [55]. These two competing aspects are referred to as the “idea problem” and the “action problem” [6,30]. Seven authors show by their results that a combination of direct and indirect ties, in the form of closely knit teams linked by sparse ties is the optimal structure for generating and producing innovation [29,30,34,35,37,46,49] with evidence that increasing the number of structural holes eventually becomes counterproductive [34].

Brokerage strategies are considered by Obstfeld [30] in the context of innovation generation and integration. Burt [5] drawing on the earlier work of Simmel [27] described the brokerage strategy of *tertius gaudens*, literally “the third who enjoys.” In *tertius gaudens* the broker co-ordinates two distant parties who are not intending to meet, or else actively maintains or exploits the two parties’ separation. *Tertius iungens,* literally “the third who joins,” was presented by Obstfeld [30] to describe the alternative strategy where the broker deliberately introduces or facilitates ties already present between two parties, either staying to further facilitate the role or stepping away. Burt [12] showed that while a *tertius gaudens* strategy across structural holes led to good ideas, it did not guarantee the wider involvement of managers which would lead to the integration of those good ideas into organisational practice. Obstfeld [30] found that a *tertius iungens* orientation, social knowledge (who knows what in the team, where to find resources) and network density were all independent predictors of innovation involvement. Lingo and O’Mahoney [29] examined the process of innovation generation and integration by observing and interviewing music producers in Nashville and showed that producers use *both* strategies at different times to benefit the network’s outcomes: using a *tertius gaudens* strategy to look for song ideas across a wide range of individuals and *tertius iungens* to resolve team differences and to provide the team with the necessary expertise to pull it all together.

Heterogeneous knowledge held by members of a network is seen as an alternative source of novel information; diverse network content being as effective in some situations as a diverse network structure. Rodan and Galunic [48] found that the range and depth of knowledge and expertise held by members of their network and their network position (measured in terms of network sparseness) were independently significant for overall performance (including innovation performance) among managers. When innovation alone was considered, network sparseness failed to be significant when knowledge heterogeneity was added to the model. As networks became denser (and the number of structural holes and brokerage potential fell), knowledge heterogeneity still remained a significant predictor of innovation.

Information environments are obviously important, a point explored by Aral and van Alstyne [35]. Their model, the “diversity-bandwidth trade-off” states that the benefit of access to novel information from a structurally diverse network (rich in structural holes) is tempered by the resultant lower flow of information possible through these weak, bridging ties (the ties’ bandwidth). Stronger ties have a broader bandwidth and so carry more information, but overall the effects of the trade-off depend on the knowledge environment the actor occupies. Strong ties with actors holding heterogeneous knowledge would be more beneficial than weak, bridging ties in a context where the state of knowledge is fairly constant. However, if the knowledge environment is rapidly advancing, with new information appearing all the time, or one in which network members have overlapping knowledge sets, actors are better served by more bridging ties. The authors find support for their model in a study of email contacts between recruiting executives.

### Knowledge brokerage

When the structural hole lies between two groups who could benefit from a transfer of knowledge, the actor in the bridging role may be thought of as a knowledge broker. Two main points were raised in the literature here. Firstly, performance of work teams is enhanced when knowledge is brokered across boundaries [28,56]. Di Marco and colleagues [28] consider a specialised knowledge broker role which they call the cultural boundary spanner in their ethnographic study of two US-Indian engineering teams. Given the rise of collaborative, multinational teams they see the potential for conflict between team members arising from differences in language, education and training. Culture here is seen as a boundary that needs to be spanned in order to increase understanding and team performance. Di Marco and colleagues found that the team with a nominated boundary spanner (an Indian expatriate that had trained in the US) was seen to clear up mismatches in the members’ knowledge systems, such as different measurement conventions and unfamiliar terms, resulting in a more collaborative team.

Secondly, there are various costs and negative sides to the brokering of knowledge [25,36-38,45,51]. When actors in a work group had to go through their leader to get information or advice from another member, that is, if the leader needed to broker the transmission of knowledge between work group members, productivity and efficiency of the work group suffered [25], team conflict increased and team viability decreased [36]. Individuals perceived a personal cost to this brokerage role which was decreased when the role was spread across the whole team [45].

### Trust

One paper in our final set of articles addresses trust and brokerage. This is a common theme in brokerage theory in the wider social network literature and is discussed at length in *Brokerage and Closure*[12]. Marsden [20] defines brokers as “intermediary actors who facilitate transactions between actors lacking access to or trust in one another.” In other words, a broker must be seen as a trustworthy intermediary by the two being brokered. Luo [26] looked at the effect of one’s network structure on one’s perceived trustworthiness within Chinese and Taiwanese technology firms. He found that actors who are go-betweens and bridges in collaborative advice networks are seen as being more trustworthy than people who are not. Social capital theory would further argue that the power and benefits of brokerage (having access to unique sources of information or resources) would be lost if the broker proved untrustworthy [12] so there is social pressure on the broker to maintain trust. Luo [26] also hypothesised that bridges may increase cooperation and general trust in the company through their perceived trustworthiness but this was not supported by the data.

## Discussion

### General

There were a range of brokerage parameters (e.g. constraint, betweenness centrality, boundary spanning ties) and methodologies (e.g. archive mining, interviews, observation) used in the studies but all were seen as valid empirical means of identifying brokers. Most of the studies that used SNA took a structural approach to brokerage by evaluating actors’ relational positions within their network and the opportunities and constraints for brokerage behaviour those positions gave [57]. In contrast, qualitative studies identified brokerage behaviour, observing how actors were using their position within the network [58]. Both approaches are based on the relationships surrounding the actors of interest and the key feature of that actor lying on the pathway between two other unlinked actors.

Another notable difference between studies was how relational data was collected. Objective methods such as counting emails or patent data, or observing actual interactions contrasted with more subjective surveys and interviews reliant on respondents’ perceptions and recall of interactions. Our decision to include widely different methods of data collection arose from the recognition that brokerage as we defined it - one actor linking two unlinked others - operates basically the same within any network. However, to ensure subjective methods were appropriately managed, all the studies were assessed for quality (e.g. checking accuracy of self reports by considering reciprocity). Low response rates for whole network surveys and studies that did not discuss how they maximised data accuracy were not included.

### The value of brokerage

A hole, says Burt [6], is an empty space and actors at the edge of a group viewing that hole may be unaware or uninterested in what is on the other side. Various group processes such as homophily (the tendency for actors with similar attributes or tasks to be linked together) and repeated interaction, strengthen the bonds between actors in a group and increase its introspection. Brokers, he argues, can see the value of bridging that hole. Burt [12] showed in his study of managers in a large electronics firm that there is a personal value to brokering. Managers whose ties bridged more structural holes were better paid, received more positive job evaluations and were more likely to be promoted, a tangible manifestation of the broker’s social capital.

The value to the network that brokers bring by crossing that hole or boundary is also considerable. They can generate innovative ideas and increase the quality of creative work. Brokerage can mediate and resolve conflict, make advice and knowledge more accessible, and can act synergistically with network cohesion and strong ties to produce environments in which collaboration can flourish.

The concept of a cultural boundary spanner [28] suggests other settings in which a boundary of language, knowledge systems or expectations may need to be deliberately mediated. Gray [15] refers to brokers as conflict resolvers in transdisciplinary teams such as those working collaboratively to translate scientific health discoveries into clinical practices. The boundary of mismatched knowledge, paradigms and experience between scientists and clinicians is best brokered by a clinician-researcher with experience of both worlds [59]. As well as pre-empting conflict by knowing what each side does not know about the other, their familiarity with both sides increases trust and collaboration.

### The costs of brokerage

The costs of brokerage are that bottlenecks in information flow may form at the broker who risks being overloaded and stressed by others’ reliance on them. There may also be a decrease in productivity as the “vision advantage” of a team high in brokerage is tempered by the cost of a dispersed focus. In addition, individuals must also bear the costs involved in maintaining bridging ties. Actors outside your cluster are likely to be different to you: involved in different work, located somewhere geographically distant or from a different profession. Since similar actors find it easier to communicate and predict one another’s behaviour, trusting ties are easier to form and maintain [60] and bridging ties require more work. Bridging ties are also harder to keep viable over time and were shown by Burt [61] to decay faster than ties to actors within one’s own cluster. Moreover, bridging ties have a short shelf life with time rendering many bridging ties and the information they broker as obsolete. Soda and colleagues [49] showed that old bridging ties in the Italian television production industry are not effective for generating innovative ideas since their usefulness is so dependent on the ever-changing context of the industry. Negative network outcomes arising from bridging or brokering ties were the potential to hoard or distort information, bottlenecks in the flow of information, and individual role overload, all resulting in a decline in overall network efficiency [62]

Rangachari’s study [47] on the positive effect of knowledge brokering on the quality of hospital coding led directly to a later paper where she suggests [56] an optimal model for such a knowledge sharing network. Her comparative models varied the three network measures of brokerage, density and hierarchy across a network made up of a management group and two or more subgroups of different professionals. She argued that the optimal network to produce performance outcomes is one high in brokerage and hierarchy with some density as seen in the high quality hospital coding teams. This model lies between the two extremes of a network in which the management group brokers across unconnected groups (high brokerage and hierarchy but low density) or one in which all teams and managers interact (low brokerage and hierarchy but high density).

Brokers are key players in the sense that they can be vital to the integrity and viability of the network. The importance of the pharmacist as a knowledge broker was revealed by Creswick and Westbrook’s study [39] of a hospital ward’s medication advice-seeking network. Likewise, identifying the bridges and brokers enabled a health promotion initiative to increase its likelihood of success in a high school network, ensuring that the message could reach right to the periphery of the network [43].

While in some circumstances brokering across structural holes may not be as efficient as being embedded in an environment rich in heterogeneous knowledge this may be dependent on the age of the network. March [63] puts forward the idea of the difference between exploration and exploitation as a firm’s strategy: exploitation uses in-house knowledge (akin here to the network rich in heterogeneous knowledge) while exploration looks elsewhere (across structural holes) for ideas. March argues that in the early stages of a firm’s (or network’s) development it may be more important to exploit the knowledge within than seek it elsewhere. This also ensures that within team ties are also fostered, building cohesion among people still learning to work with one another. Hargadon and Sutton [42], drawing on organisational memory theory [64] talk of the importance of storing and being able to retrieve knowledge acquired earlier or brought in by network members. Without an adequate process for this retrieval, the knowledge remains locked away and cannot be integrated. The authors speak of brainstorming sessions and the scheduling of deliberate opportunities to explore this mental archive among the designers; a process facilitated by their strong, cohesive ties. Cross and colleagues [65] also stress the importance of knowing “who knows what” in a collaborative network in order to maximise the use of in-house intellectual capital.

Two work group studies had contrasting results from their assessment of brokerage activity on team performance that may be explained by the nature of their knowledge contexts. Marrone and colleagues [45] looked at the benefit of boundary spanning behaviours in 31 consulting teams of MBA students who were on work experience. Susskind and colleagues [51] assessed boundary spanning behaviour in 11 project teams that included senior academics in an alliance of six major hotels and 11 hospitality based university programs. Boundary spanning improved team performance in the former and decreased performance in the latter. For Marrone’s students, this brokerage activity included advice-seeking from faculty members, and in the context of their lack of experience and advanced knowledge was effective in improving team performance. On the other hand, Susskind’s academics already had all the knowledge, expertise and experience needed within the hand-picked project team. Where a team has adequate heterogeneous knowledge held between its members, boundary spanning to outside contacts becomes a distraction which negatively affects team performance.

### Implications for healthcare

This paper argues that a knowledge of brokerage roles and their ability to improve connectivity and function across disparate groups has wide implications for healthcare. Studies in healthcare settings have shown how SNA can reveal patterns of communication between groups – both connections and gaps - and highlight key actors and areas for intervention [66]. Heng and colleagues’ [44] examination of the structure of communication between health facility management departments shows the potential a well-placed broker has to increase efficiency, and Rangachari [47] showed how bridging subgroups of professionals increased the quality of coding. The identification of the pharmacist as a key knowledge broker in Creswick and Westbrook’s paper [39] could be used to inform an intervention supporting the role. It also has implications for how medication advice-seeking should be handled after the introduction of electronic medication management systems. Will medication advice be sought as readily from a computer as from a person? Hawe and Ghali’s [43] identification of brokers among high school staff was aimed at maximising the reach and effectiveness of a health promotion intervention. This strategy of identifying bridges could be used across healthcare settings to enhance uptake of new practice guidelines or other initiatives needing to be disseminated widely and has a different focus from similar work looking at the role of opinion leaders in this process [67-69]. There is also the potential for research identifying bottlenecks in communication flows or instances of information hoarding or inappropriate gatekeeping. Future research on the enactment of brokerage roles in specific healthcare contexts [4,15] and evaluating interventions to support or introduce brokers will further inform this promising area.

Useful as SNA is to describe communication patterns and identify key actors, data collection through surveys or interviews can involve a significant time investment for already stretched clinicians and achieving a high response rate from mobile staff on rotating shifts can be difficult. This review has shown that qualitative methods such as observation and the use of documentary data are also useful and may be a preferred alternative.

## Conclusions

There are multiple lessons for health care generally, and health service management specifically. Collaborative networks by definition, seek to bring disparate groups together so that they can work effectively and synergistically together. Bridging, brokering and boundary spanning roles are crucial for bringing useful ideas from one group to another, generating innovative ideas from the selection and synthesis of diverse sources of information, and for increasing understanding and co-operation between groups. The brokerage of diverse knowledge from across a structural hole is most productive and valuable in situations where clusters have previously been isolated or introspective. Brokerage is less productive in clusters already rich in heterogeneous knowledge, and this may be a valid alternative strategy depending on the stage of the network and the intended outcomes.

Health care is a collaborative endeavour in which multiple gaps exist: between professions, departments, specialties and sites as well as the clinician-management, and indeed, the clinician-patient divide. Brokers can support the controlled transfer of specialised knowledge between groups, increase cooperation by liaising with people from both sides of the gap, and improve efficiency by introducing “good ideas” from one isolated setting into another.

There are significant costs to brokerage. Densely linked networks are more efficient at diffusing information to all their members when compared to sparsely linked groups [55]. So while a bridge across a structural hole allows information to reach actors that were previously isolated, it is not the most efficient way to transfer information. Brokers who become the holders of, or the gatekeepers to, specialised knowledge or resources can become overwhelmed by the role and so need support in order to function optimally.

## Competing interests

The authors declare they have no competing interests.

## Authors’ contributions

Research design was developed by all three authors. JL and FC reviewed papers for inclusion or exclusion. JL wrote the paper and FC, JB critically reviewed all drafts and final copy. All authors read and approved the final manuscript.

## Pre-publication history

The pre-publication history for this paper can be accessed here:

http://www.biomedcentral.com/1472-6963/13/158/prepub
